# Effects of Advanced Resistance Training Systems on Muscle Hypertrophy and Strength in Recreationally Trained Adults: A Systematic Review and Meta-Analysis

**DOI:** 10.3390/jfmk11010080

**Published:** 2026-02-16

**Authors:** Ioannis Tsartsapakis, Aglaia Zafeiroudi, Charilaos Kouthouris

**Affiliations:** 1 Department of Physical Education & Sport Science at Serres, Aristotle University of Thessaloniki, 62100 Serres, Greece; 2 Department Physical Education and Sport Science, University of Thessaly, 42100 Trikala, Greece; azafeiroudi@uth.gr (A.Z.); kouthouris@uth.gr (C.K.)

**Keywords:** drop-set training, cluster sets, advanced methods, velocity-based training, rest-pause training, tempo-controlled lifting, eccentric overload, muscle hypertrophy

## Abstract

**Background:** Advanced resistance training systems are widely used in practice, yet their comparative effectiveness for hypertrophy and maximal strength in recreationally trained adults remains unclear. This systematic review and meta-analysis evaluated whether advanced methods provide superior adaptations to traditional multiple-set training and whether specific techniques confer distinct advantages for hypertrophy and maximal strength. **Methods:** A preregistered systematic search identified randomized and non-randomized controlled trials comparing advanced resistance training systems with traditional multiple-set protocols in recreationally trained adults aged 18–45 years. Outcomes included muscle hypertrophy and maximal strength. Random-effects and fixed-effects models with Knapp–Hartung adjustments were applied, and moderator analyses examined method type, volume equivalence and proximity to failure. **Results:** Twenty-three studies met the inclusion criteria. When all outcomes were pooled, advanced systems produced a small but statistically significant advantage over traditional training (g = 0.159). Strength outcomes showed a moderate, significant benefit for advanced methods (g = 0.351), whereas hypertrophy effects were small and non-significant (g = 0.046). Rest-pause training demonstrated a modest hypertrophic advantage, while velocity-based training and eccentric overload contributed primarily to strength improvements. Drop sets, tempo-controlled training and cluster-type protocols produced adaptations comparable to traditional sets when volume and effort were matched. Across models, τ^2^ estimates were near zero, indicating minimal between-study heterogeneity. **Conclusions:** Advanced resistance training systems can be used effectively in recreationally trained adults and may offer advantages for maximal strength without compromising hypertrophy. Their hypertrophic superiority is not supported at the aggregate level, and their use should be guided by specific goals, constraints and individual preferences rather than expectations of universally greater muscle growth.

## 1. Introduction

Skeletal muscle is a highly adaptable tissue whose mass and function are regulated through mechanical, metabolic and neural stimuli. Resistance training is the primary non-pharmacological intervention for promoting muscle hypertrophy, neuromuscular performance and long-term musculoskeletal health, with evidence demonstrating improvements in metabolic function, structural integrity and physical capacity across the lifespan [1]. These adaptations arise from coordinated changes in muscle fiber morphology, excitation–contraction coupling, satellite cell activity and intracellular anabolic signaling pathways [2,3].

Beyond performance enhancement, resistance training is a central component of preventive and therapeutic exercise medicine, contributing to the management of cardiometabolic risk, sarcopenia and functional decline, particularly when integrated into long-term lifestyle interventions [4,5,6]. These health-related benefits highlight the need for optimized training prescriptions for both athletes and recreationally active adults.

Foundational recommendations emphasize training load, volume and proximity to failure as primary determinants of hypertrophy [7], while recent position stands underscore the importance of individualized programming across populations [8]. These principles underpin the design of both traditional and advanced resistance training systems in trained populations.

Research examining loading schemes shows that moderate and high loads can produce comparable hypertrophy when volume is equated, indicating that mechanical tension and motor unit recruitment can be achieved across a range of intensities [9]. Systematic reviews similarly report hypertrophy across broad load spectrums, with heavier loads favoring maximal strength [10]. Network meta-analytic evidence indicates that hypertrophy is primarily driven by effort and fiber recruitment when sets approach volitional failure [11]. These findings reinforce the central roles of mechanical tension and metabolic stress in muscle adaptation.

Traditional programming variables also influence hypertrophy and strength. Faster concentric velocities may enhance dynamic strength without consistently improving hypertrophy [12], whereas longer inter-set rest intervals support greater training volume and potentially larger hypertrophic responses [13]. Meta-analytic evidence suggests that training to failure is not required for hypertrophy, although it may affect fatigue and strength outcomes [14]. Collectively, these data indicate that mechanical, metabolic and neural factors interact to shape hypertrophic and neuromuscular adaptations.

Alongside traditional variables, advanced resistance training systems have emerged to manipulate mechanical tension, metabolic stress and neuromuscular recruitment beyond what is typically achieved with straight-set training. Techniques such as rest-pause, drop sets, cluster sets, eccentric overload, mechanical-advantage drop sets and tempo-controlled lifting have been comprehensively described [15]. These methods may influence intracellular pathways related to mTOR activation, calcium handling, metabolic stress accumulation and recruitment patterns, potentially offering distinct hypertrophic or strength-related advantages [16]. Acute evidence shows that advanced systems alter internal training load, with rest-pause and sarcoplasma-stimulating training eliciting higher metabolic stress and distinct psychoaffective responses compared with traditional training [17].

Mechanistically, advanced systems manipulate the distribution of mechanical tension, motor unit recruitment patterns and metabolic by-product accumulation within and across sets [15,16]. Rest-pause and drop-set configurations may prolong high-threshold motor unit activation, whereas cluster sets redistribute fatigue to maintain repetition quality and peak force [18,19]. Eccentric-overload and velocity-based approaches may preferentially target neuromuscular and architectural adaptations by emphasizing high force during lengthening actions or constraining velocity loss [20,21]. These distinctions provide a rationale for expecting differential effects on hypertrophy and strength while underscoring the need for rigorous comparative trials.

Advanced systems have also been proposed to increase training variety, enhance training density or elevate intensity without altering other acute variables, yet evidence regarding their superiority remains inconsistent [15].

Cluster set training has received particular attention. Cluster sets incorporate short intra-set rest intervals that facilitate partial phosphocreatine resynthesis and attenuate performance decline [18]. They may maintain higher repetition velocity, power output and mechanical tension while permitting greater volume or intensity [18]. Acute studies show reduced fatigue and preserved repetition quality [19], while chronic evidence indicates improvements in strength with inconsistent hypertrophic advantages [22]. More recent work suggests potential hypertrophic benefits through higher mechanical output, though findings remain heterogeneous [23]. Overall, cluster set effectiveness appears dependent on protocol structure, training goals and population characteristics.

Velocity-based training regulates intensity, volume and fatigue through real-time velocity monitoring [20]. However, device validity and reliability vary substantially, and accurate measurement is essential. A recent systematic review reported considerable variability in device accuracy, emphasizing the need for careful selection and calibration [24]. Thus, the effectiveness of velocity-based training depends heavily on monitoring technology quality.

Despite extensive literature, several limitations persist. Many reviews include heterogeneous populations, limiting applicability to recreationally trained adults. A recent meta-analysis found no superior hypertrophic outcomes for advanced systems compared with traditional methods [25]. The equivalence reported raises questions about whether advanced systems primarily benefit neuromuscular performance, training efficiency or psychological engagement rather than hypertrophy. This is relevant for recreationally trained adults who often adopt advanced methods expecting accelerated hypertrophy despite inconsistent evidence. The review by Fonseca et al. [25] was limited to hypertrophy, included heterogeneous training statuses and did not examine strength or moderators, underscoring the need for updated analyses evaluating both hypertrophy and strength.

Age and training status influence adaptation, with older adults exhibiting anabolic resistance and distinct neuromuscular profiles [26]. Differences between elite and recreational athletes further highlight the importance of population specificity [27]. Sex and training status modulate interference effects in concurrent training [28]. Studies combining aerobic and heavy resistance training provide insights into body composition and exercise satisfaction [29], while high-volume set configuration research shows effects on fatigue and performance without comparing advanced and traditional systems [30]

Taken together, current evidence indicates that advanced systems can modulate key drivers of muscle adaptation, yet their practical superiority over straight-set configurations remains uncertain. Existing syntheses often focus solely on hypertrophy, neglect strength or omit moderator analyses. A comprehensive synthesis integrating both outcomes and accounting for key programming variables is warranted.

Population specificity is particularly relevant for recreationally trained adults, who represent the primary users of advanced systems. This group exhibits distinct neuromuscular and metabolic profiles compared with older adults or elite athletes. Restricting the age range to 18–45 years enhances homogeneity and reduces confounding from age-related physiological changes. Focusing on healthy recreationally trained adults allows more precise characterization of advanced system effects under conditions reflecting real-world practice [26,27]. Excluding blood-flow-restriction training further isolates systems relying on mechanical tension and metabolic stress within conventional loading paradigms.

Although widely used, advanced systems are studied within heterogeneous designs, populations, volumes and proximities to failure. Some methods enhance mechanical tension or metabolic stress, while others improve repetition quality or reduce fatigue, yet translation to chronic hypertrophy or strength remains unclear. Theoretical frameworks propose increased training density, prolonged effective time under tension or enhanced motor unit recruitment, but empirical evidence is inconsistent [15]. Reviews on cluster sets suggest potential hypertrophic benefits through increased mechanical output and reduced fatigue, while repetition-tempo evidence indicates that only extremely slow tempos impair hypertrophy [21,22,31].

Given these limitations, no comprehensive and up-to-date systematic review and meta-analysis has focused exclusively on advanced systems in healthy recreationally trained adults aged 18–45 years. This population focus aligns with the predefined PICO criteria of the present review and reflects the demographic most commonly engaging in structured resistance training. This gap is notable because advanced systems are frequently used to increase training variety, manage fatigue, enhance performance or overcome plateaus. A focused synthesis is needed to determine whether advanced systems offer meaningful advantages over straight-set configurations for hypertrophy and strength and to clarify their mechanistic rationale.

The present systematic review and meta-analysis address this gap by synthesizing controlled training studies, including randomized and non-randomized trials, comparing advanced systems with traditional or alternative advanced methods in recreationally trained adults. By quantifying effects on hypertrophy and strength and examining moderators such as training status, muscle group, intervention duration, volume equivalence and proximity to failure, this work provides a mechanistically informed and practically relevant evaluation of how advanced systems influence muscle mass regulation and neuromuscular function in gym-going adults. The overarching aim is to offer a clear, updated and methodologically rigorous synthesis that can guide researchers and practitioners in understanding the specific contexts in which advanced systems may provide added value beyond traditional set configurations.

## 2. Materials and Methods

This systematic review and meta-analysis were conducted in accordance with the Preferred Reporting Items for Systematic Reviews and Meta-Analyses (PRISMA [32]) guidelines. The protocol was prospectively registered in the International Prospective Register of Systematic Reviews (PROSPERO; registration code: CRD420261282743). The PRISMA 2020 checklist is integrated within the main text, and the study selection process is detailed in Section 3.1.

### 2.1. Selection Criteria

Eligibility criteria were established a priori using the PICOT framework to ensure methodological consistency and population specificity. The review focused exclusively on healthy recreationally trained adults aged 18–45 years who were regularly engaged in structured, gym-based resistance training for a minimum of six consecutive months. This criterion was selected to ensure that the included participants had sufficient training experience to respond meaningfully to advanced resistance training systems. Studies involving untrained individuals, elite or professional athletes, older adults above 45 years, or clinical populations were excluded to avoid confounding effects related to training status, age-related anabolic resistance, or underlying health conditions.

Eligible interventions consisted of advanced resistance training systems designed to manipulate mechanical tension, metabolic stress, or neuromuscular recruitment beyond traditional straight-set configurations. These included rest-pause or myo-repetition methods, drop sets, cluster sets or rest-redistribution protocols, tempo-controlled lifting, velocity-based training, eccentric-overload or accentuated eccentric loading, and mechanical-advantage drop sets. Interventions incorporating blood-flow-restriction training were excluded to maintain mechanistic specificity and avoid conflating advanced loading strategies with occlusion-based methods.

Comparator conditions were required to involve traditional straight-set resistance training or conventional prescriptions of volume and intensity. Eligible studies were required to report at least one primary outcome related to muscle hypertrophy, assessed through cross-sectional area, muscle thickness, or lean mass, or maximal strength, assessed through one-repetition maximum (1RM) or maximal voluntary contraction (MVC). Secondary outcomes, including neuromuscular performance, psychophysiological responses, and training volume or density, were extracted narratively when available but were not included in quantitative synthesis.

Only chronic interventions lasting at least two weeks were eligible for inclusion, while acute studies were excluded to ensure that outcomes reflected true physiological adaptation rather than transient responses. The review included randomized controlled trials employing either parallel-group or crossover designs, as well as controlled intervention studies published in peer-reviewed journals. Conference abstracts, theses, case studies, and non-peer-reviewed sources were excluded.

### 2.2. Literature Search

A comprehensive literature search was conducted in PubMed/MEDLINE, Scopus, Web of Science, and SPORTDiscus from database inception to December 2025. The search strategy combined controlled vocabulary terms (MeSH) with free-text keywords related to advanced resistance training systems, hypertrophy and strength outcomes, and trained adult populations. Terms referring to advanced training methods included cluster sets, rest-pause protocols, drop sets, tempo-controlled lifting, velocity-based training, eccentric overload or accentuated eccentric loading, and rest-redistribution approaches. Outcome-related terms encompassed muscle hypertrophy, muscle growth, muscle thickness, cross-sectional area, lean mass, strength, maximal strength, one-repetition maximum, and maximal voluntary contraction. Population-related terms included recreationally trained adults, trained individuals, healthy adults, and resistance-trained participants. Boolean operators (AND, OR) were used to structure the search and ensure comprehensive retrieval of relevant studies.

In addition to database searching, the reference lists of all included studies and relevant narrative or systematic reviews were manually screened to identify additional eligible articles. The search, title and abstract screening, and full-text assessment were performed independently by two reviewers. Any disagreements regarding study eligibility were resolved through discussion or, when necessary, consultation with a third reviewer.

### 2.3. Data Collection

Data extraction was conducted independently by two reviewers using a standardized electronic spreadsheet to ensure consistency and minimize extraction bias. For each eligible study, detailed information was recorded on study characteristics, including authorship, year of publication, and country of origin, as well as participant demographics such as sample size, sex distribution, age, and training status. Intervention characteristics were extracted comprehensively, encompassing the specific type of advanced resistance training system employed, intervention duration, weekly frequency, total training volume, prescribed intensity, and proximity to failure. Corresponding details of the comparator protocols were also documented to enable accurate evaluation of methodological equivalence or divergence between conditions.

Outcome measures were extracted for all relevant hypertrophy and strength indices, including muscle thickness, cross-sectional area, lean mass, one-repetition maximum, and maximal voluntary contraction, along with the measurement techniques used in each study. When available, data on adherence, attrition, and adverse events were also recorded to provide contextual information regarding intervention feasibility and participant tolerance.

Methodological quality was assessed using the Cochrane Risk of Bias 2.0 (RoB2) tool for randomized controlled trials and the PEDro scale for controlled interventions. Publication bias was examined through visual inspection of funnel plots and Egger’s regression test when sufficient studies were available for a given outcome.

### 2.4. Statistical Analysis

All statistical analyses were performed using JASP (version 0.95.4.0). Effect sizes were calculated as standardized mean differences (Hedges’ g) with corresponding 95% confidence intervals. The initial standardized mean difference was computed as Cohen’s d using post-test means and pooled standard deviations when change scores were unavailable. Cohen’s d was defined as

$$d=\frac{\left(M_{EXP}-M_{CON}\right)}{SD_{pooled}}$$

where

$$D_{pooled}=\sqrt{\frac{\left(n_{EXP}-1\right)SD_{EXP}^{2}+\left(n_{CON}-1\right)SD_{CON}^{2}}{n_{EXP}+n_{CON}-2}}$$

To correct for small-sample bias, Hedges’ *g* was obtained by applying the standard correction factor

$$g=d\left(1-\frac{3}{4\left(n_{EXP}+n_{CON}\right)-9}\right)$$

The standard error of *g* was calculated as

$$SE\;=\;\sqrt{\left(\frac{n_{\mathrm{EXP}}\;+\;n_{\mathrm{CON}}}{n_{\mathrm{EXP}}\;\cdot \;n_{\mathrm{CON}}}\right)\;+\;\left(\;\frac{g^{2}}{2\left(n_{\mathrm{EXP}}\;+\;n_{\mathrm{CON}}\right)}\right)}$$

and the corresponding variance is

$$Var=SE^{2}$$

When studies reported multiple hypertrophy outcomes, such as muscle thickness, cross-sectional area, or lean mass, a single effect size per outcome category was extracted according to a predefined hierarchy prioritizing muscle thickness, followed by cross-sectional area and lean mass, to prevent double counting. For strength outcomes, one-repetition maximum tests were prioritized, with maximal voluntary contraction used when 1RM values were not available.

Pre- to post-intervention effects were computed using change scores when these were reported; otherwise, post-test means and standard deviations were used. When standard deviations were not provided, they were derived from standard errors, confidence intervals, or established change-score formulas. All effect sizes were corrected for small-sample bias using the Hedges adjustment.

Classical fixed-effects meta-analysis models with Knapp–Hartung adjustment for standard errors were applied. Between-study heterogeneity was quantified using τ^2^ and Cochran’s Q statistic. Across all models, τ^2^ was estimated at or near zero, indicating negligible heterogeneity. Prediction intervals were calculated to represent the expected distribution of true effects in comparable future studies.

Meta-regression analyses were conducted to examine the moderating influence of training method on pooled outcomes. The categorical moderator “method” included drop-set, eccentric-overload, rest-pause, tempo-controlled lifting, and velocity-based training, with traditional straight-set training serving as the reference category. Dummy coding was applied for all categorical predictors, and moderator significance was evaluated using Knapp–Hartung adjusted *F* statistics.

Separate meta-analyses were performed for overall pooled effects, hypertrophy outcomes, and maximal strength outcomes. Sensitivity analyses were conducted by iteratively removing individual studies to evaluate the robustness of pooled estimates. Publication bias was assessed through visual inspection of funnel plots and Egger’s regression test when at least ten studies were available for a given outcome.

## 3. Results

### 3.1. Study Selection

**Figure 1:**
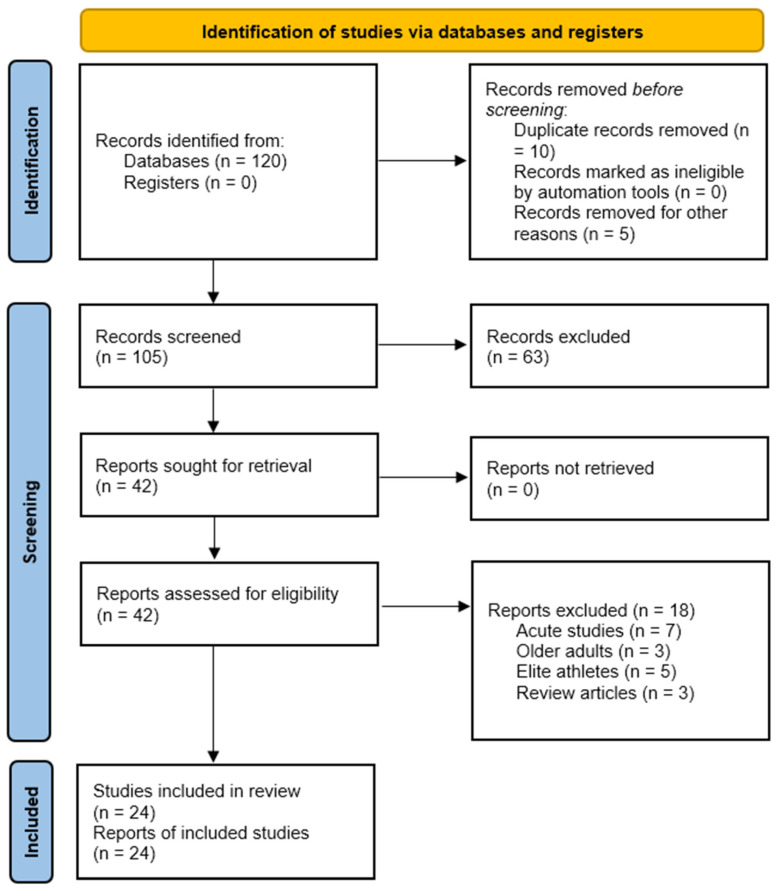
PRISMA flow diagram of study selection process.

The initial search identified 120 records through database searching. No records were retrieved from trial registers. Prior to screening, 10 duplicate records and 5 records removed for other reasons were excluded, leaving 105 records for title and abstract screening. Of these, 63 records were excluded based on relevance and eligibility criteria. Forty-two full-text reports were sought for retrieval, all of which were successfully obtained and assessed for eligibility. Eighteen reports were excluded following full-text screening due to their acute study design (*n* = 7), inclusion of older adults (*n* = 3), elite athlete populations (*n* = 5), or review format (*n* = 3). Twenty-four studies met the inclusion criteria and were included in the qualitative synthesis. The PRISMA 2020 flow diagram is presented in Figure 1.

### 3.2. Study Characteristics

A total of 24 studies were included in the qualitative synthesis, all involving recreationally trained adults and comparing advanced resistance training systems to traditional multiple-set protocols. Among the 24 included studies, 20 met the criteria for quantitative synthesis and were incorporated into the meta-analysis. The interventions examined rest-pause training, drop sets, cluster sets and rest redistribution, tempo-controlled lifting, velocity-based training, and eccentric overload methods. Reported outcomes included muscle hypertrophy, maximal strength, neuromuscular performance, and related physiological markers. Detailed study characteristics are presented in Table 1.

### 3.3. Intervention Characteristics

Intervention duration ranged from 5 to 12 weeks, with training frequencies of two to three sessions per week. Several studies equated total training volume between conditions, whereas others allowed volume to vary according to the specific demands of the advanced method. Most protocols prescribed moderate to high intensities based on percentages of one-repetition maximum. Rest-pause and drop-set methods were typically performed to momentary failure, whereas cluster-set and velocity-based protocols generally avoided failure. Full intervention details are summarized in Table 2.

### 3.4. Outcome Measures

Muscle hypertrophy was assessed using ultrasound-derived muscle thickness, MRI-based cross-sectional area, or DXA-derived lean mass. Strength outcomes were evaluated using one-repetition maximum tests for upper- and lower-body exercises or maximal voluntary contractions via isokinetic dynamometry. Additional neuromuscular and physiological measures included EMG, velocity-based assessments, and blood biomarkers. The muscle groups assessed varied across studies and included the quadriceps, elbow flexors, pectoralis major, and whole-body lean mass. All outcome measures are summarized in Table 3.

### 3.5. Risk of Bias

Risk-of-bias assessment using the ROB2 tool indicated that most studies demonstrated a low risk across domains related to the randomization process, deviations from intended interventions, missing outcome data, and measurement of outcomes. Several trials showed some concerns primarily in the domains of selective reporting or allocation procedures. Overall, the methodological quality of the included randomized controlled trials was acceptable. Full ROB2 ratings are presented in Table 4.

### 3.6. Overall Meta-Analysis (All Outcomes Combined)

**Figure 2:**
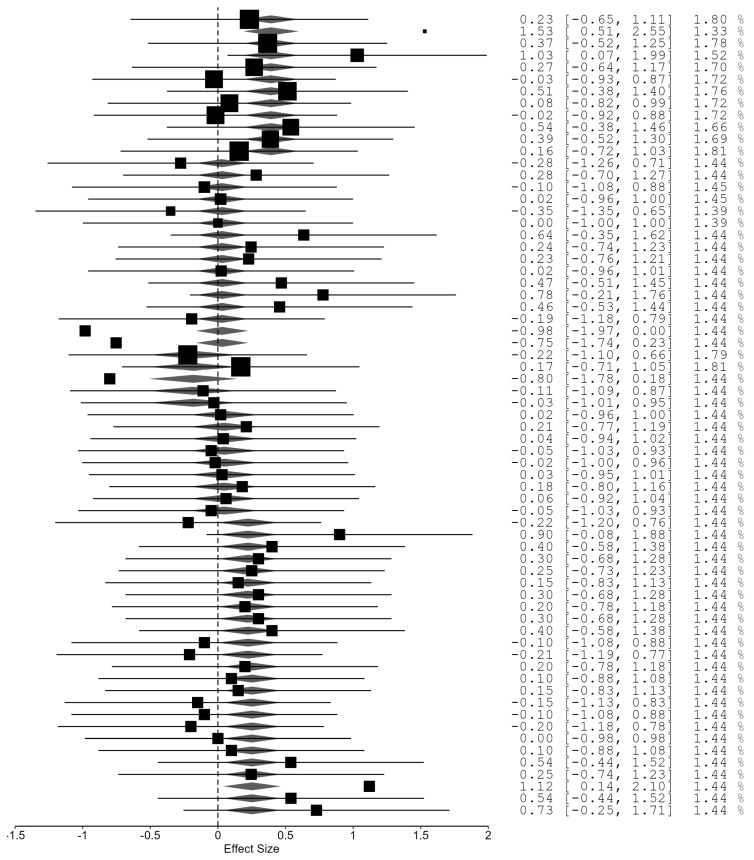
Forest plot of the overall meta-analysis pooling all strength and hypertrophy outcomes across advanced resistance training methods compared with traditional multiple-set training. Each line represents an individual effect size (Hedges’) with its 95% confidence interval, and the size of the square reflects the study weight. The diamond at the bottom depicts the pooled effect size and corresponding 95% confidence interval.

The overall meta-analysis, aggregating all strength and hypertrophy outcomes across advanced resistance training systems, revealed no residual heterogeneity, *Qe*(61) = 36.22, *p* = 0.995, with τ^2^ = 0. The pooled effect size indicated a small but statistically significant advantage of advanced methods over traditional multiple-set training, *g* = 0.159, 95% *CI* [0.066, 0.252], and *p* = 0.001. Moderator analysis identified significant between-method differences, *Fm*(5, 61) = 2.60, *p* = 0.034. Among the methods examined, rest-pause training, velocity-based training, and eccentric overload yielded the highest positive coefficients, suggesting superior overall effectiveness compared with traditional sets, whereas drop-set and tempo-controlled approaches demonstrated smaller and statistically non-significant effects. Collectively, these findings support a small but statistically significant advantage of advanced resistance training systems over traditional protocols when all outcomes are considered together, with meaningful variation in efficacy across specific methods (Figure 2).

### 3.7. Strength Meta-Analysis

**Figure 3:**
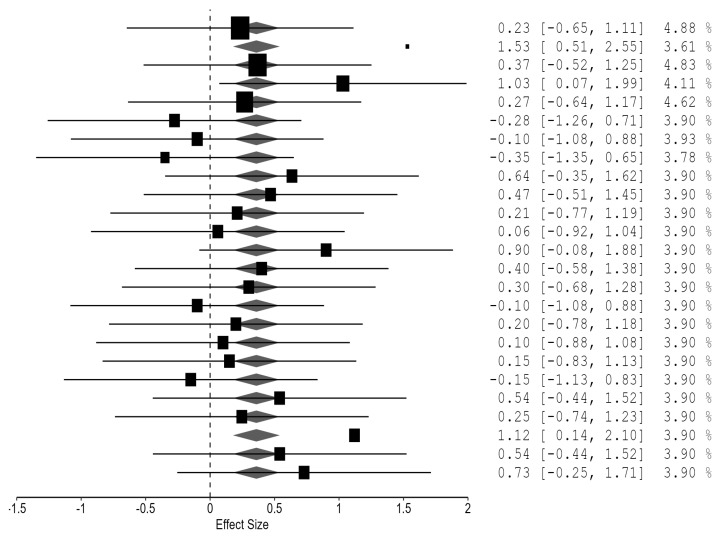
Forest plot of the strength-specific meta-analysis comparing advanced resistance training methods with traditional multiple-set training. Each line represents an individual strength-related effect size (Hedges’ g) with its 95% confidence interval, and the size of the square reflects the study weight. The diamond at the bottom depicts the pooled effect size and corresponding 95% confidence interval.

The strength-specific meta-analysis demonstrated no evidence of residual heterogeneity, *Qe*(20) = 15.29, *p* = 0.759, with τ^2^ = 0. The pooled effect size indicated a moderate and statistically significant improvement in maximal strength following advanced resistance training systems compared with traditional sets, *g* = 0.351, 95% CI [0.170, 0.533], *p* < 0.001. Moderator analysis did not reveal significant differences between methods, *Fm*(4, 20) = 1.19, *p* = 0.345. Although the rest-pause method produced the largest coefficient and approached statistical significance, no individual method clearly outperformed the others. These results suggest that advanced resistance training systems consistently enhance maximal strength relative to traditional multiple-set training, with broadly comparable effects across different methodological approaches (Figure 3).

### 3.8. Hypertrophy Meta-Analysis

**Figure 4:**
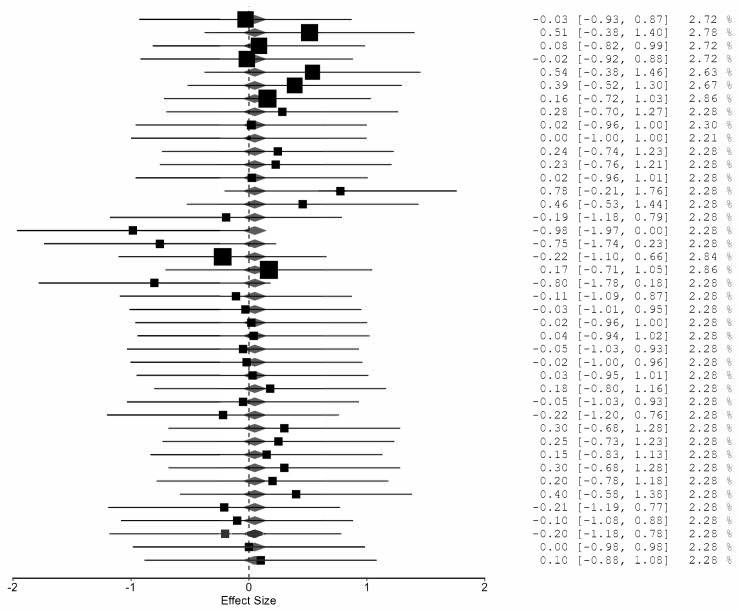
Forest plot of the hypertrophy-specific meta-analysis comparing advanced resistance training methods with traditional multiple-set training. Each line represents an individual hypertrophy-related effect size (Hedges’ g) with its 95% confidence interval, and the size of the square reflects the study weight. The diamond at the bottom depicts the pooled effect size and corresponding 95% confidence interval.

The hypertrophy-specific meta-analysis also indicated no residual heterogeneity, (*Qe*(36) = 16.02), (*p* = 0.998), with (\tau^2 = 0). The pooled effect size was small and statistically non-significant, (g = 0.046, 95% CI [−0.057, 0.149], and *p* = 0.370), suggesting no overall hypertrophic advantage of advanced methods over traditional training. Moderator analysis did not detect significant between-method differences, (Moderation *F_m_*(5, 36) = 1.27), (*p* = 0.297). Notably, the rest-pause method yielded a statistically significant positive coefficient, indicating a potential hypertrophic benefit relative to traditional sets, whereas other methods did not demonstrate significant effects. Overall, advanced resistance training systems did not collectively enhance muscle hypertrophy beyond traditional multiple-set protocols, although rest-pause training may offer a modest advantage (Figure 4).

## 4. Discussion

This systematic review and meta-analysis examined the effects of advanced resistance training systems on muscle hypertrophy and maximal strength in recreationally trained adults. When all outcomes were pooled, advanced methods produced a small but statistically significant advantage over traditional multiple-set training (*g* = 0.159). This aggregate effect was primarily driven by strength outcomes, as advanced systems yielded a moderate, statistically significant improvement in maximal strength *(g* = 0.351), whereas hypertrophy-specific effects were small and non-significant (*g* = 0.046). Taken together, these findings indicate that advanced resistance training systems confer clear advantages for strength development, while their superiority for hypertrophy in trained adults is, at best, modest and method-dependent.

### 4.1. Comparison with Previous Literature

The present results partially align with and extend prior narrative and systematic reviews on advanced resistance training methods. Krzysztofik et al. [15]) concluded that techniques such as rest-pause, drop sets, cluster sets and eccentric overload can enhance mechanical tension and metabolic stress but emphasized that evidence for superior long-term hypertrophy is limited and often confounded by volume and effort discrepancies. Similarly, a recent meta-analysis by Fonseca et al. [25]) reported no overall hypertrophic superiority of advanced resistance training systems compared with traditional multiple-set protocols, noting that differences between methods were small and often influenced by variations in training volume and effort. The current findings corroborate that view: when advanced and traditional protocols are contrasted under chronic conditions in trained adults, hypertrophy outcomes do not differ meaningfully at the aggregate level, whereas strength outcomes show a consistent advantage for advanced systems [57]. Our hypertrophy-specific findings are consistent with the general pattern reported in recent quantitative work. Fonseca et al. [25] also found that advanced resistance training systems do not lead to greater hypertrophy than traditional multiple-set protocols when training variables are comparable. The present review adds to this evidence by focusing strictly on healthy recreationally trained adults aged 18–45 years, reducing variability related to training status and age. In addition, by examining maximal strength alongside hypertrophy, we show that advanced systems provide clear strength benefits that are not visible in hypertrophy-only analyses [57]. The inclusion of moderator analyses for training method, volume equivalence and proximity to failure further clarifies the conditions under which specific advanced methods may offer distinct advantages.

The absence of an overall hypertrophic advantage does not imply that advanced methods are ineffective. Rather, the results indicate that when volume, intensity and effort are broadly matched, advanced systems produce similar hypertrophic outcomes to straight-set training in recreationally trained adults. This aligns with established evidence showing that muscle growth is primarily driven by mechanical tension, total workload and effort, which can be achieved through different programming approaches as long as these key stimuli are present [16].

### 4.2. Method-Specific Findings

Despite the absence of an overall hypertrophic advantage, meaningful differences emerged between specific advanced systems. When all outcomes were combined, rest-pause training, velocity-based training and eccentric overload displayed the largest positive coefficients relative to traditional sets, whereas drop-set and tempo-controlled methods showed smaller and statistically non-significant effects. These method-specific patterns are consistent with broader evaluations of advanced strength-training techniques. Recent work summarizing the application of advanced loading configurations in trained populations indicates that methods such as rest-pause training, velocity-based prescriptions and eccentric overload tend to produce more favorable strength adaptations than traditional straight-set training, particularly when they enable higher force production or improved repetition quality across a session [57]. This aligns with the present findings, in which these methods contributed disproportionately to the overall strength advantage observed for advanced systems. In the hypertrophy-specific analysis, the rest-pause method was the only approach associated with a significant positive coefficient, suggesting a modest hypertrophic benefit under the conditions studied.

The potential advantage of rest-pause training may be mechanistically plausible. Rest-pause configurations prolong high-threshold motor unit recruitment within a set while maintaining relatively high loads and short inter-set recoveries, thereby combining sustained mechanical tension with substantial metabolic stress [15,33,34]. This combination is thought to promote hypertrophic signaling through pathways associated with mechanical tension and metabolic stress, including mTOR-related mechanisms [7,58,59]. However, the observed effect size was small, and the number of available rest-pause trials in trained adults remains limited. Thus, while rest-pause training appears promising, current evidence supports only a cautious, method-specific advantage rather than a generalized recommendation.

Velocity-based training and eccentric overload also demonstrated relatively favorable overall effects, driven largely by strength outcomes. Velocity-based approaches individualize load prescriptions and set termination based on real-time repetition velocity, potentially optimizing neuromuscular performance by constraining fatigue and preserving repetition quality [20,60]. Eccentric overload methods, particularly those using isoinertial or flywheel devices, may enhance strength by exposing the neuromuscular system to higher forces during the eccentric phase than can be achieved in traditional isotonic lifting, which is consistent with evidence showing eccentric-emphasized training as a potent stimulus for strength and architectural adaptations [54,56,61]. In the present analysis, these methods did not yield clearly superior hypertrophy but contributed meaningfully to the strength advantage of advanced systems.

Drop sets and tempo-controlled training, in contrast, did not exhibit significant advantages over traditional sets in this population. Drop-set protocols can substantially increase training density and metabolic stress by performing sequential sets with descending loads and minimal rest, but when total volume and effort are comparable, hypertrophic outcomes appear similar to those of traditional training [38,39,41]. Prior work has suggested that drop sets may be particularly useful when time efficiency is prioritized or when the goal is to maximize metabolic stress within a constrained time frame, rather than to exceed the hypertrophic response achievable with well-structured straight-set training [41]. For repetition tempo, existing evidence indicates that moderate tempos support robust hypertrophy, whereas extremely slow tempos can reduce mechanical output and blunt gains [62,63]. The current synthesis, which excluded extreme tempo manipulations, is consistent with this pattern, showing no clear hypertrophic advantage for tempo-controlled protocols over conventional lifting in trained adults [22].

Cluster-set and rest-redistribution approaches warrant specific consideration. Recent work in trained individuals has shown that cluster sets can produce similar hypertrophy to traditional sets when volume and effort are matched, with some advantages for strength or performance maintenance depending on configuration [18,22]. In the present review, cluster-type protocols did not systematically outperform traditional sets for hypertrophy, which aligns with these data. However, cluster configurations may still be valuable for managing fatigue, preserving repetition velocity, and accommodating higher intensities or volumes over a training cycle [19]. Thus, the primary rationale for cluster use in recreationally trained adults may be related to performance and fatigue management rather than superior muscle growth per se.

Overall, the method-specific findings suggest that advanced systems are not uniformly superior. Instead, certain methods (particularly rest-pause training, velocity-based training and eccentric overload) may offer small advantages for strength and, in the case of rest-pause, possibly for hypertrophy, whereas others primarily serve to modulate training density, fatigue or perceptual responses without clearly exceeding the adaptations attainable with traditional multiple-set training [15,20,61].

### 4.3. Comparative Aggregate Effects Across Advanced Methods

Beyond the method-specific patterns described above, the aggregate effect sizes across all included studies provide additional clarity regarding the relative strength-related impact of each advanced resistance training system. Velocity-based training and eccentric-overload methods demonstrated the largest overall strength-related effect sizes, indicating that these approaches may offer superior neuromuscular adaptations when compared with other advanced configurations. Rest-pause training showed a moderate positive effect on strength, whereas drop sets, tempo-controlled lifting, and cluster-type configurations produced strength adaptations comparable to traditional multiple-set training when total volume and effort were matched. These comparative findings offer practical guidance for situations in which only one advanced method can be implemented, highlighting that methods emphasizing high force production or improved repetition quality tend to yield the most pronounced strength benefits.

### 4.4. Strength Versus Hypertrophy Adaptations

A key finding of this review is the dissociation between strength and hypertrophy outcomes. While advanced systems collectively improved maximal strength to a moderate extent, they did not confer a statistically significant hypertrophic advantage. This pattern is consistent with broader evidence demonstrating that strength gains are influenced not only by muscle size but also by neural adaptations, coordination, rate of force development and training specificity [64,65]. Techniques that preserve repetition quality, allow high intensities to be maintained, or emphasize eccentric force production may disproportionately enhance neuromuscular performance relative to hypertrophy [61].

The moderate strength advantage observed here likely reflects a combination of factors. Velocity-based and cluster-set protocols can limit velocity loss and maintain higher force and power across repetitions, potentially reinforcing neural adaptations conducive to maximal strength [18,20]. Eccentric overload methods provide high-intensity lengthening contractions that are known to enhance eccentric strength and may transfer to improved concentric performance [54,56]. Rest-pause configurations may allow additional high-intensity repetitions within a session, increasing exposure to high-threshold motor unit recruitment and thereby augmenting strength adaptations beyond those achieved with traditional set structures [33,34].

In contrast, hypertrophy appears less sensitive to these qualitative differences in set structure once total volume, intensity and effort are adequately high. This observation aligns with the principle that multiple programming strategies can converge on similar hypertrophic outcomes as long as they provide sufficient mechanical tension and overall workload [7,8]. From a practical perspective, trainers and practitioners working with recreationally trained adults might therefore prioritize advanced systems when the primary goal is strength or when specific logistical constraints (e.g., time, equipment, and fatigue management) make them advantageous, rather than expecting large additional gains in muscle size.

### 4.5. Methodological Considerations and Statistical Interpretation

An important methodological feature of the present meta-analysis is the consistency of τ^2^ estimates across models. All analyses yielded τ^2^ values at or near zero, with non-significant Q statistics, indicating an absence of detectable between-study heterogeneity. Under these conditions, the use of classical fixed-effects models with Knapp–Hartung adjustments for standard errors is appropriate, as the fixed-effects estimator provides unbiased and statistically efficient pooled effects when the assumption of a common true effect is tenable [66,67]. The Knapp–Hartung procedure further improves the accuracy of confidence intervals, particularly when the number of studies is modest [68,69].

The near-zero τ^2^ estimates across overall, strength and hypertrophy models support the interpretation that the included studies sampled a relatively homogeneous underlying effect rather than a broad distribution of heterogeneous true effects. This statistical consistency strengthens the internal coherence of the findings, as the pooled estimates are unlikely to be artefacts of large methodological or population discrepancies. At the same time, the lack of detectable heterogeneity does not preclude the existence of clinically relevant differences between specific protocols; rather, it indicates that, at the level of abstraction used in this meta-analysis, such differences were either small or not consistently expressed across the available literature [70].

### 4.6. Limitations

Several limitations should be acknowledged when interpreting these findings. First, although the overall number of included studies was moderate, the evidence available for specific advanced methods was uneven. Rest-pause, drop sets and cluster-type protocols were relatively well represented, whereas some modalities, such as eccentric overload and certain forms of velocity-based training, were supported by a small number of trials. This limits the precision of method-specific estimates and increases the risk that individual study characteristics influenced the observed effects. A key strength of this review, however, is the use of standardized effect size calculations and Knapp–Hartung adjustments, which enhance the robustness and interpretability of the meta-analytic estimates despite variability in study designs.

Second, while the review focused exclusively on recreationally trained adults aged 18–45 years to enhance population homogeneity, the included samples were still predominantly young men. Female participants and older adults were underrepresented, and sex-specific or age-specific responses to advanced systems could not be formally examined. Consequently, the generalizability of the findings to women or to individuals approaching the upper end of the age range should be considered with caution.

Third, several studies exhibited some concerns in risk of bias, primarily related to allocation procedures and selective reporting. Although most trials were judged to have acceptable methodological quality overall, inconsistent reporting of training volume, adherence, and blinding procedures remains a concern. In addition, four studies lacked sufficient statistical information for inclusion in the quantitative synthesis and were therefore summarized narratively, which may have introduced a degree of reporting bias if their true effects differed systematically from those included in the meta-analysis.

Fourth, hypertrophy outcomes were assessed using diverse methods, including ultrasound-derived muscle thickness, MRI-based cross-sectional area and DXA-derived lean mass, across different muscle groups. Although a predefined hierarchy was used to avoid double counting within outcome categories, residual measurement variability cannot be excluded. Similarly, strength outcomes encompassed various upper- and lower-body 1RM tests and maximal voluntary contractions, which, while all relevant, may differ in sensitivity to specific training methods.

Finally, the decision to exclude blood-flow-restriction training and to focus on non-occlusion-based advanced systems improved mechanistic specificity but limits direct comparison with occlusion-based methods, which are also widely used in practice. Moreover, intervention durations ranged from five to twelve weeks, and longer-term adaptations to advanced systems remain underexplored. It is possible that method-specific effects on hypertrophy or strength would diverge more clearly over extended training periods. In addition, the limited number of studies available for certain advanced methods warrants cautious interpretation, as small evidence bases may amplify the influence of individual study characteristics on pooled estimates.

### 4.7. Practical Implications and Future Research Directions

From a practical standpoint, the current findings indicate that advanced resistance training systems can be implemented effectively in recreationally trained adults without compromising hypertrophy and may offer meaningful advantages for maximal strength. Practitioners may incorporate methods such as rest-pause training, velocity-based training or eccentric overload when the goals include maximizing strength, improving training efficiency or managing fatigue while recognizing that these approaches are unlikely to produce substantially greater hypertrophy than well-structured traditional multiple-set protocols.

Future research should address several gaps. Adequately powered, volume- and effort-matched randomized controlled trials directly comparing individual advanced systems, particularly rest-pause, cluster sets, velocity-based training and eccentric overload, are needed to clarify method-specific adaptations. Studies including women and adults toward the upper end of the 18–45-year range are required to determine whether sex or emerging age-related differences influence responses. Longer-term interventions extending beyond twelve weeks, with rigorous control of training variables, would help establish whether small differences accumulate into meaningful divergences in muscle size or strength.

Further work should also include detailed reporting of adherence, perceptual responses, fatigue, recovery and adverse events to evaluate the real-world feasibility of advanced methods. Mechanistic investigations integrating neuromuscular, architectural and molecular outcomes would clarify how specific configurations influence adaptation pathways. Standardized reporting of training variables and outcome measures would enhance comparability across trials and improve the precision of future meta-analytic syntheses.

In summary, advanced resistance training systems represent a reliable and, in some contexts, advantageous alternative to traditional multiple-set training for recreationally trained adults, particularly for maximal strength. Their hypertrophic superiority is not supported at the aggregate level, and their use should be guided by individual goals, constraints and preferences.

## 5. Conclusions

This systematic review and meta-analysis indicate that advanced resistance training systems can be implemented effectively in recreationally trained adults without compromising muscle hypertrophy and with a clear, moderate advantage for maximal strength. Although certain methods, particularly rest-pause training, velocity-based training and eccentric overload, showed small method-specific benefits, advanced systems did not demonstrate meaningful hypertrophic superiority over traditional multiple-set training when volume, intensity and effort were comparable. Strength adaptations, by contrast, appeared more sensitive to the qualitative features of advanced configurations, likely reflecting their influence on neuromuscular performance, fatigue management and repetition quality.

The pattern of findings suggests that advanced methods represent viable and, in some contexts, advantageous programming options, especially when the goals include maximizing strength, optimizing training efficiency or accommodating specific logistical constraints. However, their use should be guided by individual needs and training objectives rather than expectations of universally superior muscle growth. Continued research with more diverse samples, longer intervention durations and rigorous methodological control is needed to clarify the conditions under which specific advanced systems may offer distinct long-term advantages.

## Figures and Tables

**Table 1:** Characteristics of included studies examining advanced resistance training systems versus traditional sets.

Author (Year)	Population	Advanced System	Comparator	Outcomes Reported
**Rest pause systems**				
Prestes [33]	Recreationally trained adults	Rest pause	Traditional multiple sets	Strength, hypertrophy
Enes [34]	Resistance-trained men	Rest pause	Traditional sets	Strength, hypertrophy
Karimifard [35]	Recreationally trained men	Rest pause	Traditional sets	Strength, myokines
Korak [36]	Recreationally trained adults	Rest pause	Traditional bench protocol	Strength, EMG, training volume
**Drop set systems**				
Enes [34]	Resistance-trained men	Drop sets	Traditional sets	Strength, hypertrophy
Enes [37] (RQES)	Recreationally trained men	Drop sets and related advanced methods	Traditional sets	Muscular adaptations, psychophysiology
Angleri [38]	Resistance-trained men	Drop sets, crescent pyramid	Traditional sets	Strength, hypertrophy
Ozaki [39]	Recreationally trained adults	Drop sets	Traditional sets	CSA, strength, endurance
Vilaça Alves [40]	Recreationally trained women	Drop sets	Traditional sets	Muscle thickness
Fink [41]	Recreationally trained adults	Drop sets	Traditional sets	Hypertrophy, strength
**Advanced Rest-Manipulation Systems (Cluster, Rest-Redistribution, ISR)**				
Cuevas Aburto [42] †	Recreationally trained adults	Cluster sets, rest redistribution	Traditional sets	Neuromuscular, perceptual
Vargas Molina [43] †	Resistance-trained men	Cluster sets	Traditional sets	Body composition, strength
Vargas Molina [44]	Resistance-trained individuals	Cluster sets	Traditional sets	Hypertrophy
Totó [45] †	Recreationally trained adults	Cluster sets	Traditional sets	Hypertrophy
Goto [46]	Recreationally trained adults	Cluster-type protocol	Traditional sets	Quadriceps CSA
Iglesias Soler [47]	Recreationally trained adults	Cluster-type protocol	Traditional sets	Thigh circumference
Oliver [48]	Resistance-trained men	Intraset rest intervals (ISR)	Traditional sets	Lean body mass
**Tempo controlled systems**				
Kojic [49]	Recreationally trained adults	Tempo controlled (eccentric emphasis)	Traditional sets	Hypertrophy, strength
Pearson [50]	Resistance-trained men	Tempo variation	Traditional sets	Muscle size, strength
**Velocity based training (VBT)**				
Andersen [51]	Recreationally trained adults	Velocity loss thresholds	Traditional sets	Strength, hypertrophy
Held [52]	Recreationally trained adults	Velocity based training	Traditional sets	Strength, recovery
Vasiljevic [53] †	Recreationally trained young men	Velocity based training	%1RM prescription	Neuromuscular, strength
**Eccentric overload systems**				
Norrbrand [54]	Recreationally trained adults	Eccentric overload	Traditional sets	Muscle size
Friedmann [55]	Recreationally trained adults	Computer guided eccentric overload	Traditional sets	Hypertrophy
Maroto Izquierdo [56]	Recreationally trained adults	Unilateral eccentric isoinertial	Traditional sets	Lean mass, functional outcomes

Note. Table 1 presents 25 study-arm entries derived from 24 unique studies included in the systematic review. Of these, 20 studies provided extractable quantitative data and were incorporated into the meta-analysis. Studies marked with † were synthesized narratively due to insufficient statistical information for effect size calculation.

**Table 2:** Intervention characteristics of the included studies.

Author (Year)	Advanced System	Duration (Weeks)	Frequency (Sessions/Week)	Volume Equated	Intensity (Load Prescription)	Proximity to Failure	Notes
Prestes [33]	Rest pause	6	2	Yes	80% 1RM	To failure	Standardized rest-pause protocol
Enes [34]	Rest pause, Drop sets	8	3	No	70–80% 1RM	To failure	Two advanced systems
Enes [37]	Drop sets	8	3	No	70% 1RM	To failure	Psychophysiological outcomes included
Karimifard [35]	Rest pause	6	3	Yes	75% 1RM	To failure	Myokine responses assessed
Cuevas Aburto [42] †	Cluster, Rest redistribution	8	2	Yes	70–85% 1RM	Not to failure	Intra-set rest manipulation
Angleri [38]	Drop sets, Crescent pyramid	12	3	No	70–80% 1RM	To failure	Multiple advanced configurations
Ozaki [39]	Drop sets	6	2	No	30% 1RM	To failure	Low-load drop sets
Vilaça Alves [40]	Drop sets	8	2	No	70% 1RM	To failure	Women only
Fink [41]	Drop sets	6	2	No	30% 1RM	To failure	Low-load hypertrophy protocol
Vargas Molina [43] †	Cluster sets	8	3	Yes	75–85% 1RM	Not to failure	Strength-focused cluster protocol
Vargas Molina [44]	Cluster sets	10	3	Yes	70–80% 1RM	Not to failure	Hypertrophy-focused cluster protocol
Totó [45] †	Cluster sets	6	2	Yes	70% 1RM	Not to failure	Short-duration intervention
Goto [46]	Cluster-type protocol	8	2	Yes	80% 1RM	Not to failure	Classic cluster-type protocol
Iglesias Soler [47]	Cluster-type protocol	8	2	Yes	75% 1RM	Not to failure	Thigh circumference outcomes
Oliver [48]	Cluster-type protocol	10	3	Yes	70–80% 1RM	Not to failure	Lean body mass outcomes
Kojic [49]	Tempo (eccentric emphasis)	8	3	Yes	70% 1RM	To failure	Controlled eccentric tempo
Pearson [50]	Tempo variation	8	2	Yes	60–80% 1RM	To failure	Multiple tempo conditions
Andersen [51]	Velocity loss thresholds	8	2	Yes	%1RM based on velocity	Not to failure	VL20 vs. VL40
Held [52]	Velocity-based training	6	2	Yes	Velocity zones	Not to failure	Recovery markers included
Vasiljevic [53] †	Velocity-based training	8	3	Yes	Velocity zones	Not to failure	Compared to %1RM prescription
Norrbrand [54]	Eccentric overload	5	2	No	Isoinertial device	To failure	Flywheel training
Friedmann [55]	Eccentric overload	12	3	No	Computer-controlled eccentric	To failure	High-intensity eccentric
Maroto Izquierdo [56]	Eccentric isoinertial	6	2	No	Flywheel device	To failure	Unilateral protocol
Korak [36]	Rest pause	8	2	Yes	75% 1RM	To failure	Bench press protocol

Note. Studies marked with † were narratively synthesized only due to insufficient statistical information for inclusion in the meta-analysis.

**Table 3:** Outcome measures used in the included studies.

Author (Year)	Hypertrophy Measures	Strength Measures	Measurement Methods	Muscle Groups Assessed
Prestes [33]	Muscle thickness	1RM bench press, 1RM squat	Ultrasound	Upper and lower body
Enes [34]	Muscle thickness	1RM bench press, 1RM squat	Ultrasound	Upper and lower body
Enes [37]	Muscle thickness	1RM leg press	Ultrasound	Lower body
Karimifard [35]	Muscle thickness	1RM chest press, 1RM leg press	Ultrasound, biomarkers	Upper and lower body
Fink [41]	Muscle thickness	12RM triceps, 1RM arm curl	Ultrasound	Elbow extensors/flexors
Angleri [38]	Muscle thickness	1RM lower-body lift	Ultrasound	Lower body
Ozaki [39]	Cross-sectional area	1RM arm curl	MRI	Elbow flexors
Vilaça Alves [40]	Muscle thickness	None	Ultrasound	Elbow flexors
Vargas Molina [44]	Muscle thickness	1RM squat	Ultrasound	Quadriceps
Goto [46]	Muscle thickness	1RM squat	Ultrasound	Quadriceps
Iglesias-Soler [47]	Thigh circumference	1RM squat	Tape measure	Quadriceps
Oliver [48]	Lean body mass	1RM squat	DXA	Whole body, lower body
Kojic [49]	Muscle thickness	1RM squat	Ultrasound	Lower body
Pearson [50]	Muscle thickness	1RM squat, 1RM bench press	Ultrasound	Upper and lower body
Andersen [51]	Muscle thickness	1RM squat, MVC, velocity metrics	Ultrasound, velocity tracking	Quadriceps
Held [52]	None (recovery markers)	1RM squat, 1RM bench row, 1RM deadlift	Blood markers, velocity tracking	Upper and lower body
Norrbrand [54]	Cross-sectional area	MVC	MRI, isokinetic dynamometry	Quadriceps
Friedmann [55]	Muscle thickness	Peak torque	Ultrasound, isokinetic dynamometry	Quadriceps
Maroto-Izquierdo [56]	Lean mass	MVC, 1RM leg press	DXA, isokinetic dynamometry	Lower body
Korak [36]	None (EMG, volume)	1RM bench press	EMG, 1RM testing	Upper body

**Table 4:** Risk-of-Bias (ROB2) assessment for included randomized controlled trials.

Author (Year)	Randomization Process	Deviations from Intended Interventions	Missing Outcome Data	Measurement of Outcomes	Selection of Reported Results	Overall Risk of Bias
Prestes [33]	Low risk	Low risk	Low risk	Low risk	Low risk	Low risk
Enes [34]	Some concerns	Low risk	Low risk	Low risk	Some concerns	Some concerns
Enes [37]	Some concerns	Low risk	Low risk	Low risk	Some concerns	Some concerns
Karimifard [35]	Low risk	Low risk	Low risk	Low risk	Low risk	Low risk
Fink [41]	Some concerns	Low risk	Low risk	Low risk	Some concerns	Some concerns
Angleri [38]	Some concerns	Low risk	Low risk	Low risk	Some concerns	Some concerns
Ozaki [39]	Low risk	Low risk	Low risk	Low risk	Low risk	Low risk
Vilaça Alves [40]	Some concerns	Low risk	Low risk	Low risk	Some concerns	Some concerns
Vargas Molina [46]	Low risk	Low risk	Low risk	Low risk	Low risk	Low risk
Goto [46]	Some concerns	Low risk	Low risk	Low risk	Some concerns	Some concerns
Iglesias Soler [47]	Some concerns	Low risk	Low risk	Low risk	Some concerns	Some concerns
Oliver [48]	Some concerns	Low risk	Low risk	Low risk	Some concerns	Some concerns
Kojic [49]	Low risk	Low risk	Low risk	Low risk	Low risk	Low risk
Pearson [50]	Low risk	Low risk	Low risk	Low risk	Low risk	Low risk
Andersen [51]	Low risk	Low risk	Low risk	Low risk	Low risk	Low risk
Held [52]	Some concerns	Low risk	Low risk	Low risk	Some concerns	Some concerns
Norrbrand [54]	Some concerns	Low risk	Low risk	Low risk	Some concerns	Some concerns
Friedmann [55]	Some concerns	Low risk	Low risk	Low risk	Some concerns	Some concerns
Maroto Izquierdo [56]	Low risk	Low risk	Low risk	Low risk	Low risk	Low risk
Korak [36]	Some concerns	Low risk	Low risk	Low risk	Some concerns	Some concerns

Note. Risk of bias was assessed using the Cochrane Risk of Bias 2 (ROB2) tool across five domains.

## Data Availability

Data available on request due to restrictions (e.g., privacy, legal or ethical reasons).

## Abbreviations

The following abbreviations are used in this manuscript:

RT	Resistance Training
RM	Repetition Maximum
1RM	One Repetition Maximum
RCT	Randomized Controlled Trial
PRISMA	Preferred Reporting Items for Systematic Reviews and Meta-Analyses
ES	Effect Size
CI	Confidence Interval
SD	Standard Deviation
VL	Velocity Loss
VBT	Velocity-Based Training
DOMS	Delayed Onset Muscle Soreness
RoB	Risk of Bias
MD	Mean Difference
SMD	Standardized Mean Difference
g	Hedges’ g
τ^2^	Between-study variance
Qe	Cochran’s Q statistic for residual heterogeneity
I^2^	Inconsistency index
